# Subscapularis Tendon Repair Using Transtendon Double-Pulley Technique With All-Suture Anchor

**DOI:** 10.1016/j.eats.2025.103799

**Published:** 2025-08-05

**Authors:** Jidong Song, Zhaopu Jing, Tian Lei, Lihong Fan

**Affiliations:** Department of Sports Medicine and Pediatric Orthopeadics, The Second Affiliated Hospital of Xi'an Jiaotong University, Xi'an, China

## Abstract

The management of subscapularis tears presents a significant challenge because of the complexity of the rotator cuff injury. The conventional suture technique, employing either a single-row or double-row stitch, has been shown to have inherent limitations, including inadequate mechanical stability, compromised blood supply, and the potential for postoperative stiffness. We propose a transtendon double-pulley technique. This technique involves the placement of an all-suture anchor through the subscapularis tendon, a dynamic double-pulley in the footprint area to construct a dynamic tension system through cross-stitching. This technique integrates the biological advantages of tendon fixation with the mechanical adaptability of the dynamic pulley system, offering a repair option for subscapularis tears.

Subscapularis tendon tears, accounting for 15% to 30% of all rotator cuff injuries, are increasingly recognized as critical contributors to shoulder dysfunction and anterior instability.^1^ Unlike supraspinatus tears, subscapularis injuries often present diagnostic and therapeutic challenges as the result of their deep anatomical location and complex fiber orientation. Arthroscopic repair has become the gold standard, yet achieving robust tendon-to-bone healing remains problematic, with reported retear rates ranging from 10% to 25%, depending on tear size and surgical techniques.^2^

Current arthroscopic approaches predominantly rely on single-row or double-row suture configurations.^3^ Single-row fixation, although technically straightforward, demonstrates limited contact area and uneven pressure distribution, leading to greater failure rates in larger tears. Double-row techniques improve footprint coverage but require extensive dissection and may compromise vascularity. The "suture bridge" method enhances biomechanical strength by linking medial and lateral anchors, yet excessive suture tension often precipitates tendon cut-through, particularly in osteoporotic bone. Recent innovations, such as the "lasso-loop" and "pulley-link" techniques,^4^^,^^5^ attempt to balance load distribution, but their reliance on peripheral sutures still risks inadequate grasping of the tendinous portion.

In this study, we describe a transtendon double pulley (TTDP) technique that synergizes transtendon all-suture anchor with dynamic pulley technique. The TTDP method involves one medial anchor placed at the articular margin with transtendon sutures, capturing >80% of tendon thickness, and a lateral pulley anchor, creating a self-adjusting tension system through crisscross suture routing. This dual mechanism achieves 3 critical advantages: First, the transtendon sutures enhance grasping force. Second, the dynamic pulley redistributes stress across the footprint. Third, preserved vascular channels between suture strands may accelerate biological healing.

## Surgical Technique

### Patient Setting and Diagnostic Arthroscopy

General anesthesia is administered. The patient is positioned in the lateral decubitus position with 30° to 40° of posterior rotation.^6^ The shoulder is abducted 70° and flexed 15° anteriorly using a balanced suspension system, and the patient's trunk is protected by an occipital cushion and bean bag system.

Three portals are established: the posterior viewing portal, anterior working portal, and anterosuperalateral portal.^7^ The 30° arthroscope is used to access the joint cavity via a posterior approach, with a small incision made 1 to 2 cm medial to the posterior posterolateral corner of the acromion and 2 cm below. The anterior working portal positioned within the rotator interval is then created under direct visualization and is positioned midway between the coracoid process and the anterolateral acromion. The extent and morphology of the subscapularis tear and its precise location are assessed using the Lafosse classification. The condition of the bony surface is examined to determine whether the bone needs to be trimmed or cleaned. In addition, the long head of the biceps tendon also is evaluated. In case of any pathology of this tendon, tenotomy or tenodesis is performed.

### Tissue Release and Bone Bed Preparation

After confirming the subscapularis tear, the subscapularis tendon is released using the 3-sided release technique.^8^ The torn edge is shaved to remove poor-quality tissues. The thick fibrous tissues accompanying synovitis cover the rotator interval and subscapularis tendon are resected for clear visualization of the subscapularis tendon. For larger tears, the coracohumeral ligament and adhesions to the anterosuperior margins of the subscapularis tendon is released to facilitated better mobilization. The arm is positioned in maximal external rotation and 70° to 90° forward flexion to expose the lesser tuberosity. The lesser tuberosity footprint is debrided and decorticated with a burr to expose bleeding bone, ensuring optimal biologic healing.

### Subscapularis Tendon Repair Using Transtendon Double-Pulley Technique

Using a specialized curved guide device (Delta Medical, Beijing, China), the subscapularis tendon is made a controlled splitting using the fish-mouth dilation technique through the anterior portal. The first 1.8-mm V-lock all-suture anchor (Delta Medical) is placed through the guide device under direct visualization, ensuring sutures pass through the tendon substance without requiring a suture hook or scorpion. The second 1.8-mm V-lock all-suture anchor (Delta Medical) is then placed into the exposed footprint beyond the tendon in the case of small tear. The second anchor could also be transtendon placed in the case of larger tear.

**Fig 1:**
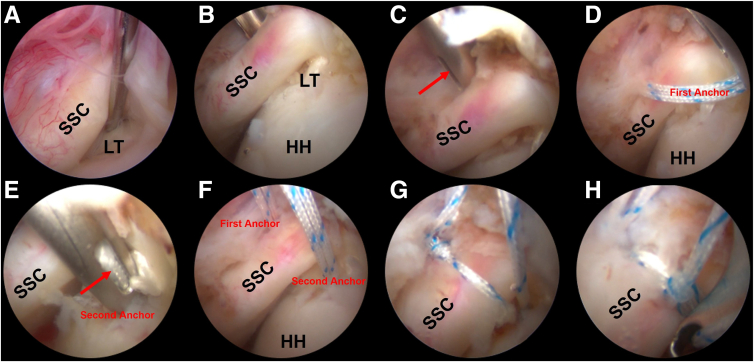
Subscapularis tendon repair using trans-tendon double-pulley technique with all-suture anchors (Left side). (A) The subscapularis tendon injury viewed from the posterior portal and the lesser tuberosity is prepared. (B-C) Fish-mouth tendon dilation technique is used to make a controlled longitudinal splitting in the subscapularis tendon at the footprint. (D) The first anchor is placed. (E-F) The second anchor is placed at the lesser tuberosity, it can also be placed trans-tendon. (G-H) Sutures are tied with a double-pulley technique. The patient is placed in lateral decubitus position and posterior portal serves for observation and anterior portal serves for operation (the anterosuperalateral portal is used as the auxiliary operation portal). Red arrow, fish-mouth tendon expansion technique. Lateral decubitus position, 30° posterior tilt, 70° abduction; viewing: 30° arthroscope via posterior portal. (HH, humeral head; LT, lesser tuberosity; SSC, subscapularis tendon.)

**Fig 2:**
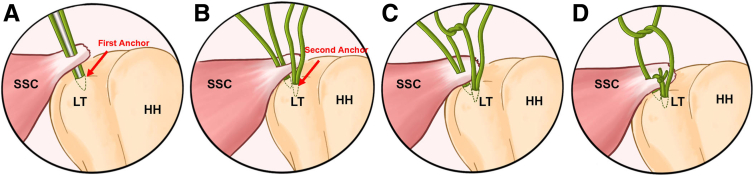
The schematic diagram of transtendon double-pulley technique for subscapularis repair (Left side). (A) First anchor is placed at the lesser tuberosity footprint after a controlled longitudinal splitting is made in the subscapularis tendon using the fish-mouth technique. (B) The second anchor is placed using the same technique; this anchor can be directly placed into the lesser tuberosity or transtendon. (C) The first knot the of pulley is tied in extracorporeal manner and then pulled into the joint by pulling the other limbs. (D) The other pair of limbs are then tied using the nonsliding knotting technique to finish the final double-pulley configuration. The patient is placed in lateral decubitus position and posterior portal serves for observation and anterior portal serves for operation (the anterosuperalateral portal is used as the auxiliary operation portal). (HH, humeral head; LT, lesser tuberosity; SSC, subscapularis tendon.)

One suture pair from each of the anterior and posterior anchors is retrieved through the anterior portal. One suture limb from each anchor is chosen to be coupled in a double-pulley configuration.^9^ The limbs are tied in extracorporeal manner using multiple half-hitches on alternating posts. Traction on the other 2 limbs advances the knot into the joint onto the anterior surface of the subscapularis tendon. These limbs are then tied using the nonsliding knotting technique. The extra length of the suture limbs is finally removed using the arthroscopy scissors (Figs 1 and 2).

### Postoperative Care and Rehabilitation

The shoulder should be immobilized using a shoulder brace, as indicated by the patient's specific situation, to prevent repair failure resulting from postoperative strenuous activities.^10^ Postoperative braking is performed for a duration of 6 weeks to enable immediate movement of the hand, wrist, and elbow. Beginning in the first postoperative week, patients can engage in light shoulder joint activities and avoid excessive weight-bearing. From the second to the fourth week postoperatively, the range of motion is gradually increased to ensure that the subscapularis muscle gradually regains function. Regular follow-ups at 1, 3, and 6 months after surgery are crucial for monitoring the repair effect and adjusting the rehabilitation program as necessary. If deemed necessary, imaging tests will be performed to confirm the healing of the tendon.

## Discussion

Subscapularis tears represent one of the most complex pathologies in rotator cuff injuries, posing a significant challenge in clinical management. Although arthroscopic advancements have improved the feasibility of minimally invasive techniques, existing methods remain constrained by critical limitations. Traditional single-row sutures have inadequate mechanical stability, whereas double-row configurations, despite enhancing contact area, concentrate suture tension at the bone-tendon interface.^11^ This stress concentration predisposes the repair to complications such as suture cutting or anchor loosening, often termed the "cheese-wiring effect."^12^ In addition, conventional suturing techniques risk compromising vascular integrity, failing to address the unique anatomical and biomechanical demands of subscapularis repair.

To overcome these challenges, we describe a composite technique integrating tendon fixation with dynamic tension modulation using the all-suture anchor. The innovation lies in 2 key components: First, a tendon-penetrating all-suture anchor is deployed to enhance grip strength. By employing a "red-red zone" suturing pathway, this approach ensures full-thickness tendon penetration while preserving vascular supply. Second, a dynamic pulley system is engineered to optimize stress distribution. The synergistic interplay between tendon-penetrating anchors, which provide initial stabilization, and the dynamic pulley system, which shares functional loads, establishes a "point-to-surface" hybrid fixation mechanism.

Although the technique involves a learning curve as the result of the intra-articular line-crossing procedure in all-arthroscopic approaches, its fundamental advantage lies in the integration of established arthroscopic principles. By systematically combining conventional anchor placement and suture management protocols, the procedure maintains technical accessibility. With structured preoperative planning and standardized training, surgeons familiar with basic arthroscopic skills can effectively master this methodology to achieve consistent clinical outcomes.^13^ Although the TTDP technique demonstrates biomechanical superiority in load distribution, 3 inherent limitations warrant cautious implementation. First, the dynamic pulley mechanism inherently increases suture complexity. Second, although transtendon anchors enhance footprint compression, tendon splitting during anchor placement may transiently impair vascular perfusion in the central tendon region. Third, although all-suture anchors reduce bone damage, their cyclic displacement resistance remains lower than PEEK (polyether ether ketone) anchors in osteoporotic bone.^14^^,^^15^

Advantages and disadvantages of this technique are discussed in Table 1. This technique harmonizes biomechanical optimization with minimally invasive principles, offering a promising solution for subscapularis repair. With ongoing refinements in surgical technology and accumulating clinical experience, such integrative approaches provide an option for the treatment of subscapularis tear.

**Table 1:** Advantages and Disadvantages

Advantages
Biomechanically, anchors that penetrate the tendon provide a stronger grip than traditional surface sutures.
Dynamic tensioning of the double pulley system avoids stress concentrations.
All-suture anchors require low bone mass and are suitable for osteoporotic patients.
Preserves the vascular supply to the tendon, potentially promoting healing.
Disadvantages
May not work for large tears or severely retracted tendons.
Intraoperative suture management is challenging for less experienced surgeons.
More research is needed to validate the long-term effects.

## Disclosures

All authors (J.S., Z.J., T.L., L.F.) declare that they have no known competing financial interests or personal relationships that could have appeared to influence the work reported in this paper.

## Supplementary Data

Video 1 The patient is in the lateral decubitus position (the lateral decubitus position: 30° posterior tilt, 70° abduction; viewing: 30° arthroscope via posterior portal). Three portals (posterior viewing, anterior working, and anterosuperolateral) are established. The subscapularis injury is explored, and the lesser tuberosity footprint is debrided and decorticated. Two 1.8-mm V-lock all-suture anchors are placed transtendon through the guide device using the fish-mouth technique. One suture limb from each anchor is chosen for a double-pulley configuration, and the other 2 limbs are pulled to advance the knot onto the anterior surface of the subscapularis tendon. The nonsliding knotting technique is used to tie the limbs, and the extra length of the suture limbs is removed with arthroscopy scissors.
